# Not all brains are created equal: the relevance of individual differences in responsiveness to transcranial electrical stimulation

**DOI:** 10.3389/fnsys.2014.00025

**Published:** 2014-02-24

**Authors:** Beatrix Krause, Roi Cohen Kadosh

**Affiliations:** Department of Experimental Psychology, University of OxfordOxford, UK

**Keywords:** inhibition, excitation, transcranial electrical stimulation, individual, responsive, efficacy

## Abstract

A current issue in the research of augmentation of brain functions using transcranial electrical stimulation (tES) is the diversity and inconsistency in outcome results. Similar studies often report different results, depending on the parameters and tasks used. Such inconsistencies have led to significant doubts about the efficacy of the method in the broader scientific community, despite its promising potential for patient recovery and treatment. Evidence on the large variability in individual cortical excitability and response to tES suggests that stimulation may affect individuals differently, depending on the subject’s age, gender, brain state, hormonal levels, and pre-existing regional excitability. Certain factors might even lead to the reversal of polarity-dependent effects, and therefore have crucial implications for neurorehabilitation and cognitive enhancement. Research paradigms may have to be refined in the future to avoid the confounding effects of such factors.

## Introduction

Transcranial electrical stimulation (tES) in its various forms (anodal vs. cathodal transcranial direct current stimulation (tDCS); transcranial random noise stimulation (tRNS); and transcranial alternating current stimulation (tACS)) has become a highly popular research tool to enhance a wide range of typical, as well as atypical cognitive patterns of behavior (Miniussi et al., 2008; Brasil-Neto, 2012; Cohen Kadosh, 2013; Krause and Cohen Kadosh, 2013). A multitude of studies include healthy participants, patients with behavioral and neuropsychiatric disorders, as well as brain damage or neurological conditions. The external modulation of cortical excitability aims to induce beneficial changes in cortical efficiency and functioning, and thereby enhance plasticity, which subsequently improves the outcome of the training or testing variable in question. The enhancement of synaptic plasticity in the stimulated area is thought to increase the area’s processing efficiency, which supports learning and/or recovery (Cramer et al., 2011). However, the diversity of different interactions between deficits and functional systems in different individual populations, as well as the resulting potential individual differences in tES effects has not been disentangled yet. In fact, differences at the individual level of regional brain function and anatomy may lead to profoundly different outcomes. The current idea is that excitatory tES methods, such as anodal tDCS and tRNS enhance cortical excitability (Nitsche and Paulus, 2001; Terney et al., 2008), whereas cathodal tDCS decreases cortical excitation (Nitsche et al., 2003). However, while this might be a generally accepted idea, the real pattern seems more complex.

We have previously suggested that the optimal balance between cortical excitation and inhibition (E/I balance) differs between individual brain areas and subjects and therefore the application of, for instance, anodal tDCS may lead to fundamentally different results in an individual with high regional excitability (i.e., anodal tDCS will lead to overexcitation and non-optimal performance), whereas the same stimulation in a different brain regions with different E/I levels, or in an individual with lower excitation may be more beneficial (Krause et al., 2013). The optimal excitability level would then be at the tip of an inverted-U shaped function of excitation/inhibition and behavior. In line with this hypothesis, researchers have now discovered that experimental populations tDCS research can almost be split into two separate groups: responders and non-responders (López Alonso et al., 2014). The study was based on a previous study that used transcranial magnetic stimulation (TMS) to the motor cortex to investigate cortical excitability in the form of motor evoked potentials (MEPs) in a sample of 56 individuals at various different time points (Hamada et al., 2013). Instead of averaging across the whole group, as is most commonly done in brain stimulation research, Hamada et al. (2013) tracked the patterns of MEP amplitudes per subject across the eight time points. The result was that the individual responses were highly variable and when averaging across all subjects, the average result was nearly zero. More recently, López Alonso et al. (2014) observed a similar pattern using tDCS. A cluster analysis pointed to a subset of 55% of the subjects that not only failed to show an increase, but instead even a slight decrease in TMS-elicited MEP amplitudes in response to the stimulation, which was significantly distinguishable from the 45% of subjects that showed the expected increase. Similarly, another study has shown that increasing the level of excitation by increasing stimulation intensities of tRNS and tACS can reverse excitation to inhibition (Moliadze et al., 2012). There are methods to assess changes in cortical excitability, such as magnetic resonance spectroscopy (MRS) or TMS, but there are also methods to affect inhibition and excitation. For example, paired associative stimulation (PAS), deep brain stimulation (DBS) and direct current stimulation (DCS) in rodents, can improve the interpretation of data. In humans, MRS is an especially valuable research technique in this regard, as it is a noninvasive *in vivo* magnetic resonance imaging (MRI) method that reliably assesses total concentrations of GABA and glutamate in a predefined voxel (Mekle et al., 2009), typically between 1.5 × 1.5 × 1.5 cm^3^ and 3 × 3 × 3 cm^3^, which makes it possible to estimate E/I in the to-be-stimulated brain area. Such findings are highly relevant to the field of tES research and we will later discuss a variety of influences on cortical excitability that may be responsible for the drastic individual differences in responses to the stimulation previously described. The identification of such confounding variables may improve tES research and analysis strategies in the future and allow for better controlled design and application of tES in research and potentially in the future also in clinical settings.

## Neurotransmitter balances

The brain’s main excitatory and inhibitory neurotransmitters, glutamate and GABA, respectively, are strongly involved in learning and experience-dependent plasticity (Trepel and Racine, 2000; Ge and Dani, 2005). For example, regional GABA levels decrease with learning in the domain associated with the stimulated brain region (e.g., motor learning in M1) (Floyer-Lea et al., 2006). Moreover, the higher the observed learning increment, the steeper the GABA decrease in response to anodal tDCS (Stagg et al., 2011). Such a reduction can in turn facilitate long-term potentiation (LTP), which allows for cortical reorganization (Hess and Donoghue, 1994) and the authors suggest that the responsiveness of an individual’s regional GABA system to the stimulation is related to their learning capacity.

Assuming that an increase in cortical excitability is beneficial for learning, we should also be aware of some of its negative consequences. Overexcitation of the cortex (i.e., the excessive release of glutamate), leads to excitotoxicity and cell death (Faden et al., 1989; Belousov, 2012). Excessive GABAergic inhibition, however, prevents LTP and reduces neuronal output (Mcdonnell et al., 2007). Enhanced inhibition is therefore associated with higher network stability but also reduced cortical plasticity (Hess and Donoghue, 1996). Accordingly, a fine balance in the interaction between excitation and inhibition is required to optimize the efficiency of information transfer in the brain (Turrigiano and Nelson, 2000; Bavelier et al., 2010). For tES application, this means that there is a certain dose-response relationship that interacts with pre-existing baseline levels that are currently unknown to the experimenter. Besides other confounding factors in tES research that we will discuss later, this interaction could explain the observed individual differences in experimental outcomes and the large variability in the current literature.

So far, researchers have mainly been concerned with extreme abnormalities in excitability (for instance epileptic patterns of brain activity), and have used it as an exclusion criterion for tES experiments. It is important to note that other neurotransmitter systems also interact with cortical excitability and therefore abnormal neurotransmission in those may equally moderate the effects of tES and potentially the subjects’ health. For instance, elevations in extracellular serotonin are associated with increased excitability induced by anodal, and surprisingly also by cathodal tDCS (Nitsche et al., 2009). Therefore, to avoid these confounds in experimental work, we generally recommend to exclude individuals with psychological or psychiatric problems, as well as individuals taking medication that influences hormone or neurotransmitter systems.

Individual differences in pre-existing neurotransmitter levels and in cortical efficiency are also reflected in brain activity, as measured by functional magnetic resonance imaging (fMRI), such that baseline levels of glutamate and GABA are associated with regional activity levels. For example, GABA concentrations measured by MRS correlate positively with γ oscillation frequency, which reflects inhibitory activity, and are inversely related with functional activity in the cortex (Muthukumaraswamy et al., 2009). This means that inhibition can be expressed in the strength of γ oscillations measured by electroencephalography (EEG) or magnetoencephalography (MEG), and that the task-related blood-oxygenated level dependent (BOLD) response decreases with elevated inhibition. Similarly, the baseline GABA concentration predicts the properties of the activation-dependent hemodynamic response function (HRF), such that higher baseline inhibition is related to lower activity (Muthukumaraswamy et al., 2012). Furthermore, task-dependent activity in several different cortical and subcortical areas is associated with glutamate levels in the brain area in question but also in remote areas that are heavily connected. However, the direction of the relationship between activity (low vs. high) and task demands is modulated by pre-existing glutamate levels (low vs. high) (Falkenberg et al., 2012).

These results demonstrate that common findings in brain activation studies can be reasonably well explained by local concentrations of baseline glutamate and GABA levels. Moreover, individual differences in pre-existing neurotransmitter levels cause research subjects to respond differently to external modulation of E/I. For example, a subject with high initial inhibition may never reach a similar level of regional cortical plasticity as a subject with low inhibition. In turn, with two different groups of individuals showing opposing effects in response to tES, the outcome effect will be reduced, or even regress the mean of the whole sample towards zero.

## Current application

Given the large number of options available in the selection of tES parameters, the effects on the individual subject’s cortical excitability and tissue may be very specific and extremely variable across a whole sample. For instance, there are sharp contrasts in outcomes observed using different current strengths, such that 1 and 2 mA of A-tDCS achieve different outcomes on cognitive tasks. One study reports reduced reaction times with prolonged but not shorter stimulation periods at 2 mA, whereas reaction times increased with longer stimulation times at 1 mA (Teo et al., 2011). Reaction times were therefore similarly low under short periods of 1 mA and longer periods of 2 mA. Similarly, 2 mA of C-tDCS over the motor cortex can even flip the intended inhibitory effect on MEPs achieved at 1 mA into cortical facilitation (Batsikadze et al., 2013). Such reversal effects suggest that more (in terms of both intensity and duration) is not necessarily better and there is a fine line between the optimal and accidentally impairing current application.

Several different variations of tES are available, whereby the underlying neurobiological mechanisms are better understood for some than for others. For instance, the user can decide whether to excite a region in one hemisphere and inhibit the same region in the other, or he can place one of the electrodes on an area with minimal or no interference (e.g., the vertex, forehead, cheek or arm). It is currently unclear, which option is ideal for which purpose (for a first step see Moliadze et al., 2010). One of the major reasons for this lies in the principle of interhemispheric inhibition. The two hemispheres work in concert to produce behavioral output and damage to an area in one hemisphere may unleash unprecedented activation of the same area in the other hemisphere (Cramer et al., 1997; Zimmerman and Hummel, in press). In the presence of brain damage or dysfunction for example, the contralateral hemisphere often tries to compensate for the loss and therefore responds with atypical patterns of activity (see Johnston, 2009). The particular pattern is known to the tES user, however, such that the prediction of tES effects is specific to the choice of parameters.

The effect of left anodal tDCS (cathode attached to the forehead) on resting state activity in a prefrontal network indeed demonstrated increases in functional connectivity to the same area in the right hemisphere, whereas connectivity to other areas within the same hemisphere was reduced (Park et al., 2013). The authors hypothesized that the behavioral results found in cognitive tES studies may be based on the changes in interhemispheric connectivity and that different placements of the cathode may have caused fundamentally different results. Such effects may be similar or different for different brain areas and between the two hemispheres. Additionally, different subject populations might respond differently to such effects, depending on the pre-existing interhemispheric connectivity patterns (e.g., see for elderly Cabeza, 2002). Given this fact, the question about the optimal stimulation method for a given purpose is critical.

In tACS the current alternates between the cathode and the anode at a fixed frequency (Zaghi et al., 2010) and it is known to modulate brain oscillations. Its beneficial effect on cognition or behavior has not yet been fully established, and has even been found to impair perceptual processes in certain cases (Brignani et al., 2013). From DBS we know that the stimulation frequency also leads to fundamental differences in the effectiveness of the treatment of for instance motor disorders, such as Parkinson’s disease (PD) (see McConnell et al., 2012). Despite the fact that DBS (with its implanted electrodes) has a different mechanism of action, it demonstrates how varying the stimulation parameters can successfully direct the output effects of a stimulated cortical network. There is now also first evidence for the effectiveness of tACS in modulating Parkinson-related brain oscillations (Brittain et al., 2013). Similarly, the effect of the different stimulation frequencies used in tACS often depends on external factors, such as lighting conditions within the testing room (Kanai et al., 2008). In this case the same stimulation parameters in a well-lit room may differ from the ones in a room under darker conditions during perceptual processing. The authors point to an interaction between ongoing cortical oscillations in the cortex and the applied current frequency. Accordingly, the subject’s current cortical excitability will interact with the stimulation. Where tACS has a variety of different frequencies that can be freely chosen (e.g., α, β and γ waves) tRNS also has different frequency settings, that are mostly split into full-spectrum, high-frequency (Hf-) and low-frequency (Lf-) tRNS. Under certain conditions, tACS has been shown to induce stronger excitability increases than full-spectrum tRNS (Moliadze et al., 2012), but when tRNS conditions are directly compared, Hf-tRNS induces stronger excitability than Lf-tRNS (Terney et al., 2008). Again, it is important to note that there are few experiments available that compare different parameters of tES within the same study and often the outcome measure is an excitability variable (mostly in the motor domain), rather than a cognitive or behavioral outcome. This means that the current knowledge might be restricted to very specific experimental conditions and it is unknown whether these effects are generalizable across domains and testing conditions.

There is a large variety of tES applications with its excitatory vs. inhibitory modulation (anodal and cathodal tDCS), excitation through noise induction (tRNS) and the modulation of cortical oscillations (tACS), as well as possible parameters including frequency range, current strength and electrode positioning interact with ongoing regional excitability of the cortex. The problem is that the experimenter is usually unaware of the excitability levels and these might differ under different experimental conditions and might be particularly sensitive in perceptual domains.

## The research design

In addition to individual differences in biological substrates, variations in study design can have a striking impact on the outcomes of tES studies. For example, daily tDCS leads to greater excitability changes than second daily application (Alonzo et al., 2012) such that a more sensitive neural system may accumulate higher excitability over several sessions. In addition, it is crucial to assess long-term effects of improvements and potentially impairments, as these eventually determine the success of the intervention. Some have already demonstrated long-term positive effects (e.g., Reis et al., 2009; Cohen Kadosh et al., 2010b; Snowball et al., 2013), whereas most studies do not perform such follow-up testing. Another question is at what point in time an improvement in the testing variable will be visible. Many studies test and evaluate performance during the stimulation (e.g., Bolognini et al., 2010; Weiss and Lavidor, 2012), whereas others also compare pre- and post-measures (e.g., Dockery et al., 2009; Snowball et al., 2013). The quality of the effect may be different in such cases and before tES can be applied in clinical settings, the evolution of performance change should be monitored to find the optimal time for training assessments.

## The initial brain state

An important but hard to control factor in research is the brain state of the individual subject. Silvanto et al. (2007) point out the importance of subject factors, such as fatigue and wakefulness, attention, intoxication and the habituation to the presented task material. These and others can be potential confounders that can even flip polarity-dependent effects of tES into the opposite polarity. For example, state-dependent effects associated with baseline brain activity can be related to resting α-band power, which has been showed to modulate the threshold for excitability probed by TMS (Romei et al., 2008). Similarly, using tACS with its ability to entrain cortical oscillations, the stimulation frequency has been showed to interact with ongoing brain activity. The highest increase in motor cortical excitability at rest was achieved using β-tACS (20 Hz), whereas the highest excitability levels during motor imagery were observed during θ-tACS (5 Hz) (Feurra et al., 2013). The authors attribute the effect of θ-tACS to the underlying use of working memory processing during the imagery task, whereas β stimulation is thought to correspond to the natural cortical response during rest.

Using a neural adaptation paradigm, Silvanto et al. demonstrated that less active neuronal populations respond more strongly to TMS than more active ones (Silvanto et al., 2008). Further studies extended this finding to high-level cognition and the parietal lobes (Cohen Kadosh et al., 2010a). Furthermore, the experimental manipulation of cortical excitability responds in a similar way, such that preconditioning the cortex with A-tDCS causes repetitive TMS (rTMS) to be inhibitory, whereas C-tDCS preconditioning reverses subsequent rTMS effects to cortical excitation (Lang et al., 2004; Siebner et al., 2004). The effects of the initial brain state are even visible across different tasks. Motor cortical excitability could be reduced by A-tDCS, and increased by C-tDCS to M1 during a cognitive task, compared to the same stimulation during rest (Antal et al., 2007). However, cortical excitability was reduced by both A-tDCS and C-tDCS when engaging in a motor task, compared to during rest. In this study, A-tDCS therefore only increased cortical excitability during rest, but flipped the effect to inhibition during cognitive, and even more so during motor engagement. In contrast, C-tDCS led to a slight excitability decrease at rest but a sharp decrease during motor processing, while it increased excitability during cognitive processing. The authors concluded that areas that are not involved in the cognitive task at hand become deactivated, while the reduction in excitability during the motor task is more likely to be associated with muscle fatigue. It is therefore apparent that ongoing neuronal activation interacts with different types of stimulation to modulate cortical excitability and behavioral responses. The effects of experimentally uncontrolled influences on the brain seem so profound that they have the potential to even flip intended inhibition to excitation (and *vice versa*) and are therefore not consistent with the current idea of polarity-specific tDCS. Currently, such baseline cortical activity factors are unknown variables in tES research. Experimental instructions and procedures may bias the brain state of subjects to respond to the stimulation in a certain way, confounding the desired outcome.

## The individual brain

The situation is further complicated by individual variations in head and tissue morphology. Different head sizes and tissue thicknesses might cause different current distributions and require different current strengths to achieve the same current flow (Bikson et al., 2012). For example, depending on where on the head the electrodes are placed, the stimulation can be more focal than in other configurations, which may be related to the orientation of neurons and the current flow applied and how the current propagates along the tissue connections (Neuling et al., 2012). Individual morphologies of cortical gyri and sulci also affect the pattern of the current flow (Datta et al., 2012). The same stimulation design can therefore lead to large differences in the induced current and the resulting electric field, due to brain and body morphological differences (Datta et al., 2012; Truong et al., 2013). The resulting individual differences in the strength of the induced electric field effects on neuronal activity and E/I will therefore be fundamentally different. As observed in experiments applying different intensities of current (e.g., Batsikadze et al., 2013), an intended excitation can flip to inhibition in some subjects but not in others. This in turn will affect both physiological and behavioral effects negatively.

Furthermore, depending on the task applied under stimulation (especially using cognitive tasks), subjects may recruit different brain regions for the same task depending on their stage of brain and cognitive development and due to the individual strategy use (Rivera et al., 2005). The consequence of this is that one might stimulate an area that is currently not involved in the processing of the task (i.e., the “wrong” area for the task at hand based on previous fMRI studies in different populations), which will eventually not benefit the individual’s abilities and add further noise to the experimental results.

Similarly, the interaction between brain areas might differ across individuals, e.g., due to differences in the strength and efficiency of network connections. Since tES has been found to affect whole networks rather than just the stimulated region in isolation (Keeser et al., 2011; Zheng et al., 2011), it is possible that by enhancing brain functioning at one point in the network, subsequent network areas might be negatively affected, such that the outcome is disadvantageous (Brem et al., 2014). For example, the increased cortical excitability may lead to reduced inhibition to a subsequent area, such that this area will produce excessive output and impair behavioral functioning. It has indeed been found that stimulation of frontal areas can improve certain cognitive aspects while interfering with others, while stimulating parietal areas reverses the pattern (Iuculano and Cohen Kadosh, 2013). Considering this possibility, individuals with certain neurological vulnerabilities, young and old individuals, as well as patients with brain abnormalities or damage can be expected to respond differently to the same type of stimulation. For instance, the behavioral effects of tES on elderly compared to younger participants seem to be reversed and hemisphere-dependent (Ross et al., 2010, 2011). The anticipation of tES effects should therefore never be generalized from one group to another, but should instead carefully explored to prevent a null result, or even an accidental induction of cognitive impairment.

## The developing and aging brain

The brain is not static. It changes continuously across the lifetime and, along with these changes, occur changes in behavior and how the individual responds to stimuli in the environment. During development, a certain relative balance between excitation and inhibition defining functional properties of the cortex is established and eventually maintained throughout later stages of life (Turrigiano and Nelson, 2004). However, evidence from animal research suggests that early experiences shape the coupling of excitatory and inhibitory neural activity and thereby affect cortical plasticity. Still, the initial interactive activity is subject to change and refinement across the course of postnatal development (Dorrn et al., 2010) and E/I balances may therefore guide the timing of developmental critical periods of plasticity for experience-dependent learning (Hensch and Bilimoria, 2012). Early experiences and E/I interactions may therefore determine the later responsiveness to tES.

Research using TMS demonstrated how age-related differences in cortical excitability affect the speed of signal transduction in motor pathways. Motor responses are slowed in elderly compared to younger adults (Smith et al., 2009), which is associated with age-related changes in intracortical inhibition. This leads to a decline in the functional modulation of corticospinal activity (Fujiyama et al., 2012). Similarly, the aging individual also has to face cognitive slowing, which has been associated with a weakening of white matter connections between different cortical areas but also a decline in structural gray matter, whereby the pace of change differs by the structure or area (Raz et al., 2005; Jackson et al., 2012).

Along with such changes in brain structure, regional neurotransmitter balances change as well, affecting experience-dependent plasticity (Hess and Donoghue, 1996). For example, GABAergic receptor distribution changes from early childhood to the early teenager years and then again during older age (Pinto et al., 2010). More specifically, there is an age-related decline in GABA levels that can be observed in the elderly brain using MRS (Gao et al., 2013). Glutamate availability has also been found to decrease in the aging brain in rodents (Mcentee and Crook, 1993). An observed loss in NMDA receptors (Wenk et al., 1991) (in rats and monkeys) may be responsible for such changes and may also impact the capacity to form LTP. With such changes and shifts in E/I, experience-dependent plasticity in the cortex changes across the lifetime (Hensch et al., 1998; for a comprehensive review on the role of spike timing-dependent plasticity in plasticity, see Caporale and Dan, 2008). Interestingly, the artificial reduction of GABAergic signaling can restore some of the age-related decline in learning in rodents (Lasarge et al., 2009). Therefore, the external modulation of GABA using tES may also lead to beneficial behavioral effects in the elderly but it is unclear how the type and dosage of the stimulation affects elderly individuals differently from younger age groups. The evidence on regional GABA and glutamate concentrations, as well as on the effects of tES in elderly populations is currently extremely scarce.

In summary, along with the continuous brain development and age-related changes in structure and function, we can expect changes in E/I balance across the lifespan, which are currently under-investigated. The interactions between tES and E/I balance are therefore even less predictable than in healthy young adults. This is due to the fact that the research on *in vivo* assessments of GABA and glutamate in developing and aging human samples and the use of tES in these groups is still in its infancy and there is little available evidence at this point.

## Circadian rhythm

Circadian influences, such as sleep and time of the day have been found to affect cortical excitability, such that TMS-probed intracortical inhibition was found to decrease throughout the day (Lang et al., 2011). Moreover, with more time staying awake, especially after sleep deprivation, motor cortical excitability gradually increases along with an increase in EEG θ waves, which is commonly observed with prolonged wakefulness (Huber et al., 2013). This sleep-dependent increase in cortical excitability has critical implications for subjects’ sleeping patterns prior to stimulation. Sleep deprivation may enhance the risk for seizure activity, especially in combination with repeated sessions of tES, which by itself increases excitability (Alonzo et al., 2012). Such combination could accumulate cortical excitability to potentially harmful levels in susceptible participants. A variety of different psychological and neuropsychiatric disorders involve abnormal circadian rhythms or deficient sleep patterns and some can already be distinguished on the basis of MRS-measured GABA and/or glutamate concentrations at a group level (e.g., Goto et al., 2009; Yoon et al., 2010; Rojas et al., 2013). Careful screening procedures should therefore be applied to monitor potential pre-existing abnormalities in E/I.

## Hormonal levels

**Figure 1:**
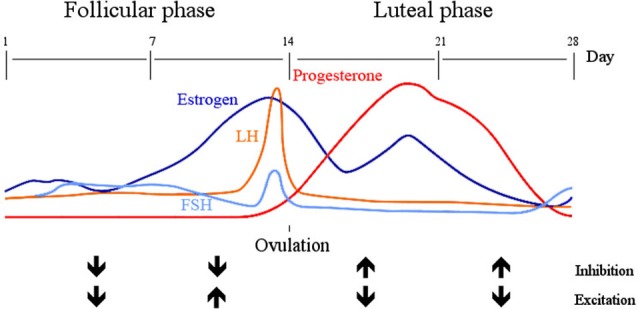
**Inhibition and excitation across the menstrual cycle.** The early follicular phase (day 1 –the start of menstruation—until day 7) is characterized by low levels of estrogen and progesterone, during which both inhibition and excitation are low. With an increase in estrogen from day 7–14, luteinizing hormone (LH) and follicular stimulating hormone (FSH) peak and stimulate ovulation on day 14. During this late follicular phase inhibition is low and excitation high, which reverses in the following 14 days (early luteal phase). Estrogen, in particular estradiol is now moderately high but progesterone peaks.

Another source of variation is related to hormonal levels, which fluctuate substantially more in women than men, such that some studies exclude females completely from their research on cortical excitability to reduce the noise (e.g., Alonzo et al., 2012). Two main phases in the menstrual cycle can be distinguished: the follicular phase, characterized by rising levels of estrogen and low levels of progesterone, and the luteal phase, which starts with ovulation and is associated with moderate levels of estrogen and high levels of progesterone. Cortical inhibition, as probed by TMS and measured by MEPs measured is enhanced and simultaneously excitability reduced, during periods of higher progesterone levels (i.e., the luteal phase) (Smith et al., 1999). Furthermore, cortical excitation is relatively low during the first half of the follicular phase (including the menstruation period), which is characterized by low levels of both progesterone and estradiol in particular, but then excitability increases in the second half of the follicular phase, when progesterone is still low but estradiol peaks (Smith et al., 2002). Excitation is then decreased again, and inhibition increased during the luteal phase, with rising progesterone and estradiol levels. Progesterone therefore seems to drive the increase in cortical inhibition, whereas estradiol increases excitability. This is supported by a study using MRS to assess GABA concentrations in the primary visual cortex, which appeared lower during the luteal than the follicular phase in healthy women and GABA was inversely related with both estradiol and progesterone levels (Epperson et al., 2002). However, since the study did not concurrently measure levels of glutamate, no inferences can be made on the E/I balance, as those might counteract or dominate GABA levels differentially during different phases of the cycle. Moreover, in the study that measured cortical excitability using TMS (Smith et al., 2002), the follicular phase was subdivided into an early and a late phase due to the peaking levels of estradiol in the second half, whereas the MRS study investigated the follicular phase as a whole, which may have failed to capture the measurement during the rising levels of estradiol. Instead, the researchers subdivided the luteal phase into early and late, the two halves of which show little difference in their respective estradiol and progesterone levels (Epperson et al., 2002). This might additionally have affected the interpretation of GABA levels throughout the cycle. For future studies it will be useful to subdivide both phases equally and inspect all four time points. In a different study, glutamate concentrations in the medial frontal cortex were found to be significantly lower during the luteal than the follicular phase (Batra et al., 2008). Since both studies investigated different brain areas, it is hard to draw conclusions but it is likely that assessments of the ratio of glutamate and GABA will confirm the findings of previous TMS studies that relative inhibition is increased during the luteal phase, and reduced during the follicular phase (see Figure 1 for a summary of the current results in relationship with estrogens and progesterone).

For a more complete picture of global brain excitability, Harada and associates investigated three different brain areas (left frontal cortex, lentiform nuclei and cingulate cortex) using MRS and found that only in the lentiform nuclei and the left frontal cortex GABA levels were decreased during the luteal compared to the follicular phase but not in the anterior cingulate cortex (Harada et al., 2011). This means that E/I might additionally depend on the interaction between hormonal fluctuations and local brain regions. To make things more complex, smoking is another possible noise variable in E/I balance, as GABA levels have been found to differ across the menstrual cycle. Specifically, GABA is higher during the follicular than the luteal phase in nonsmoking women (Epperson et al., 2005). Furthermore, there was no difference between smoking and non-smoking men and their GABA levels were similar to women in the luteal phase. In contrast, smoking women in the follicular phase showed slightly but not significantly reduced levels of GABA, compared to all other groups. No differences in GABA concentrations were found after 48 h of abstinence in the smokers. Despite the fact that an individual’s lifestyle may affect E/I and induce additional noise into tES experiments, long term smoking behavior in this case might even stabilize GABA concentrations (although causal inferences from these study results are not possible).

A rapid change in E/I balance due to hormonal fluctuations has been associated with neurological conditions, which already serve as exclusion criteria and/or control variables in tES research. These are for instance migraine, epileptic seizures, but also premenstrual mood disorders (i.e., during periods of high cortical inhibition) (for a more detailed discussion on biological alterations, see Finocchi and Ferrari, 2011). Despite the increasing evidence on excitability differences due to hormonal changes, this type of information is not by itself informative about the efficiency of the information transfer in the brain and the degree of capacity for plastic changes.

## Driving and predicting plasticity

By transiently enhancing plasticity, we attempt to induce favorable long-lasting changes that allow for increased experience-dependent learning. However, there is also an optimal balance between plasticity (i.e., the flexibility of synaptic connections to change according to experience) and stability (keeping the system balanced). Critical periods of development are characterized by enhanced levels of brain plasticity because learning during such periods is crucial to shape the cortical networks for later efficient processing (stability). Stability is necessary to make the network cost-efficient in terms of energy expenditure and thereby provide constant and predictable patterns for the preferred output (Knudsen, 2004). Strengthening pathways in the “wrong” way during such a period of high plasticity levels can cause unwanted and irreversible (stable) changes during cortical development (Knudsen, 2004). Therefore, caution is needed when artificially enhancing plasticity, as behavioral changes in the wrong direction may be difficult to reverse.

Stability, in terms of long-term adaptation to plasticity-induced training effects may therefore be more crucial to tES than previously anticipated. This means that we should not aim for the maximum cortical excitability, but instead for the *optimal balance* between plasticity and stability in terms of E/I. Not only does the E/I balance determine the flexibility of a network and hence the functioning of the brain area (Knudsen, 2004; Murphy et al., 2005), but additional compensatory mechanisms also regulate the net output of the system. While Hebbian learning drives experience-dependent plasticity, compensatory mechanisms are required to maintain the stability in a homeostatic way and thereby maintain a fixed set point of firing rates (Turrigiano and Nelson, 2004). This process is called homeostatic plasticity. The previously discussed flip in excitatory and inhibitory effects after polarity-dependent tDCS is another example of homeostatic plasticity. Some authors have suggested that the cortex uses this flip to maintain a functionally useful range of cortical excitability, which subsequently affects the potential for plasticity (Lang et al., 2004; Siebner et al., 2004). They explain that pre-existing excitation and inhibition determine the net effect of TMS and whether its action is excitatory or inhibitory. Such pre-existing E/I levels may be directly related to differences in the state of the brain (e.g., alertness, attention or familiarity with a task and neuronal populations), and thereby interact with the applied stimulation (Silvanto et al., 2007). Therefore, in the presence of elevated levels of excitation, TMS or tES effects may be incapable of inducing further excitation and instead may suppress cortical activity. Neurorehabilitation would therefore be naturally limited.

Given a history of successful cognitive and clinically symptomatic improvements achieved by tES, the question is how strongly these homeostatic mechanisms are affected by the stimulation. This question has been neglected in tES research so far and is worth serious investigation. If tES is affected by homeostatic plasticity, there might be an upper level of improvement we cannot exceed and the question is whether excessive stimulation will lead to a ceiling effect or whether it reverses the effects to the worse. The latter possibility would reflect our previous hypothesis of an inverted-U shape of cortical excitability and behavioral outcome (Krause et al., 2013). Beyond a certain point the stimulation will start causing impairments rather than further improvements. Another question is if homeostatic set points differ across individuals and whether the range of possible plastic changes inducible by tES is similar in each individual. Furthermore, we also have to take into account that this set point may change with development and aging. Dose-response experiments monitoring changes, especially impairments in behavioral and cognitive outcomes, are therefore of high importance (e.g., Teo et al., 2011; Moliadze et al., 2012; Brignani et al., 2013).

In practice, these compensatory mechanisms are likely to occur over an extended period of time and may therefore not be immediately measurable in changes on behavioral tasks or symptom outcomes used during experiments. However, changes induced by homeostatic plasticity can even counteract Hebbian learning effects (Turrigiano and Nelson, 2004), which may moderate the effects of tES greatly. This again has important implications for rehabilitation, especially in epilepsy, where an unintended increase in excitability may have severe health effects.

## How to deal with the variability

The wide variety of options for tES parameters paired with the multitude of individual differences in pre-existing neurotransmitter levels makes the evaluation of tES research results as a whole difficult. Guleyupoglu et al. (2013) stress the importance of current dose for the outcome of the study. For example, they define tES dosage in terms of the parameters of the electrodes, including the size, number, shape, position and composition, as well as the waveform in terms of intensity and the general form of the waves administered, the pulse shape (wherever relevant), amplitude, width, polarity and repetition frequency of the current waves, the number of sessions and the inter-session interval. For research purposes, as well as for the generalization of results it is important to always report the exact parameters used, as the current flow and the resulting induced electric fields depends on these parameters (Peterchev et al., 2012). In severe clinical cases when the financial means are available, electrode shapes and sizes can even be custom designed to better control and enhance tES effects but this method requires structural brain scans (MRI) and neuronavigation for the production and fitting of the stimulation (Tecchio et al., 2013). Individualized tES with more focal effects (high-definition tDCS) may become more feasible in the future, with the development of more automated and less time-consuming methods for the prediction of current flow (Datta et al., 2012; Huang et al., 2012; Edwards et al., 2013).

One possible solution to specifically target the individual regional E/I balance is to assess GABA and glutamate levels in the voxels of interest using MRS and thereby determine the directionality of the current polarity/parameters to optimize E/I. Similarly, tACS has been shown to have particularly long-lasting effects on cortical excitability and cognition when the stimulation frequency is tuned to the endogenous cortical firing frequency, as assessed by EEG (Neuling et al., 2013). This way, tES applications can be individually tailored. However, the high costs of neuroimaging may not always be feasible and more knowledge of the ideal balance required. Many of the current experiments exclude women due to the hormonal fluctuations and the influence on cortical excitation but if we want to eventually affect brain and behavior of the general population using tES, we must explore the relationship between the menstrual cycle and E/I further. The same applies to developing and aging populations. In the case of hormonal influences, blood measures of hormone levels may give indications of relative cortical excitability levels in women. However, more evidence is required to substantiate the evidence on the relationship between E/I and hormonal interactions.

Another solution, which might be a somewhat crude indicator, is individual variability in behavior. If E/I is related to brain oscillations and metabolic responses, which again are associated with behavioral response patterns, behavioral performance may distinguish at least extremely elevated or reduced levels of E/I. While behavioral performance does not capture the entire E/I variance or other factors that we mentioned here, it may still serve as a useful additional controlling factor for the variety of influential factors discussed here.

## Discussion

Evidence stemming from noninvasive brain stimulation studies suggests that there are separate subgroups of experimental subjects that differentially respond to stimulation. Specifically, up to half of them respond with reductions in excitability in response to the stimulation, whereas the other half responds, as expected, with increases in measures of excitability (Wassermann, 2002; Hamada et al., 2008; López Alonso et al., 2014). Such subgroups may have diminished many of the expected beneficial effects of tES in the past and identification of the type of responder before the application of tES would substantially help the outcome analysis and interpretation of tES effects. In order to achieve successful clinical intervention, this information is crucial for the user. We believe that the biological determinants of the subject response outcome depend on neurotransmitter balances, in particular glutamate and GABA, as their interaction defines the E/I balance in the area that is to be stimulated. The baseline in E/I balance might be differently skewed in each individual, such that some start off with higher relative excitation, whereas others have relatively low regional excitability. This will subsequently have differential effects on the capacity for the induction of plastic changes and therefore lead to different outcomes in experiments where each subject receives the same treatment. This would explain why in some cases up to half the sample responds to the enhanced excitability, whereas the same enhancement is disadvantageous in others (López Alonso et al., 2014). As Pavlov hypothesized more than 50 years ago, different personalities underlie different ratios between excitation and inhibition and therefore produce different behavioral outcomes (for a discussion, see Strelau, 1997). How these differences in cortical E/I balance arise is currently unknown, but it has been found that siblings show similar responses to brain stimulation, which suggests that there is either a heritable factor biasing the E/I balance, or that stimulation responses are similar in siblings due to some common morphological properties of their skulls and cortices**** (Wassermann, 2002).

For reasons of baseline neurotransmitter system activity, tES might lead to different results in different individuals. This can be due to the discussed factors and potential pre-existing vulnerabilities, as well as structural differences in cortical gray and white matter, skull and tissue thickness. The effects of tES can therefore be binary (effect vs. no effect), or they can show in varying degrees of the effect or even in negative effects. The outcome of the result does not always allow for inferences on the underlying mechanism, such that the uncertainty in current research interpretations is concerning. The degree of the response to tES may also vary with time of the day, the time point of menstrual cycle in women, environmental testing conditions, and general preexisting levels of neurotransmitter balances in the brain. These may further be influenced by medication or lifestyle preferences, such as smoking (as well as other methods of intoxication) or sleep patterns.

We would also like to note that our view implies that polarity-dependent effects of tES are not always straightforward and predictable. The common view that A-tDCS is generally excitatory and C-tDCS inhibitory has been challenged, as discussed here, and can be seen as a relatively crude average outcome. For example, the polarity-specific effect of tDCS depends on the organization, morphology and orientation of cortical neurons to the incoming current (Bikson et al., 2004; Kabakov et al., 2012; Rahman et al., 2013). Due to the curvature of an axon, there is always a combination between excitatory and slightly stronger inhibitory activity, such that the sum between these determines the net output (Kabakov et al., 2012).

For these reasons, it is possible that the biological processes underlying tES effects are even more complex than we suggest here. Despite the extensive research on inhibitory and excitatory effects of the current, the effects may not solely be explained by E/I. For instance, a subtle change in excitation and inhibition induced by weak current can alter network dynamics by simply changing the pattern of E/I. These changes are non-linear and the size of the effect depends on the current state of the network, affecting the rate and timing of neuronal firing (Reato et al., 2010). This means that even small simultaneous changes in the levels of excitation and inhibition can lead to a different outcome within the dynamics of the network.

Cellular tissue studies can provide more direct evidence for our idea in the future. However, investigating cellular E/I interactions in human higher-level cognition is currently not feasible. In order to fully understand the effects of tES on plasticity-behavior relationships, we have to understand the resulting formation of LTP and long-term depression (LTD) by the actions of NMDA and AMPA receptor activity (Huganir and Nicoll, 2013). Examining individual differences at the receptor level in humans is *in vivo* unfortunately is not feasible at the moment. It is also important to note that there is quite a leap from the interpretation of current effects at the receptor level in cell tissue and human behavioral studies. The bridge between molecular and human research consists of computational modeling of the current flow through different tissue configurations (for a comprehensive review see e.g., Bikson et al., 2012), which is a useful tool to understand current effects. In order to understand and predict the exact effects of tES (with its different types and parameters) we need to understand the cellular, broader cortical (e.g., regional interactions between brain areas) and behavioral effects of tES and how these are linked together. This link may be far simpler for lower-level skills, such as motor or perceptual brain functions, but more complex for higher-order cognitive abilities, including attention, working memory and arithmetic.

A potential problem with the majority of the evidence from tES research on cortical excitability is that it is all performed in the motor domain and may therefore not be generalizable to other behavioral and cognitive domains. For instance, the motor cortex may respond in fundamentally different patterns to other areas (e.g., in the prefrontal cortex). For example the observed reduction in inhibition associated with anodal tDCS and motor learning (Floyer-Lea et al., 2006; Stagg et al., 2009) stands in sharp contrast to the beneficial effect of cathodal inhibition on certain frontal cognitive functions (Weiss and Lavidor, 2012). In some domains cortical inhibition appears to be more beneficial than in others, especially considering cognitive functions involving attentional focus or the inhibition of irrelevant material, such as in the latter case. Therefore, one should be cautious about generalizing across domains.

MRS can be used to quantify concentrations of glutamate and GABA in different areas. As a measure of E/I balance, these quantifications are already used to distinguish healthy from psychiatric populations (e.g., Eichler and Meier, 2008; Yoon et al., 2010; Kubas et al., 2012). The addition of this neuroimaging technique or similarly EEG/MEG to measure oscillations, tES studies for the predetermination of E/I levels in potential subject pools would be very cost intensive. Nevertheless, we believe that this might clarify tES results and foster our understanding of both functional brain neurochemistry and tES methodology in the future.

## Conclusion

Here we summarized the most well-known currently unpredictable factors influencing cortical excitability and plasticity and how the interaction of individual differences with the available multitude of stimulation parameters may influence the effects of noninvasive, plasticity-inducing electrical stimulation at the individual level. We conclude that the simple perception of tES polarity-specific neuronal modulation is an oversimplification of the complex effects and that the effects are currently far less predictable than assumed in the majority of the scientific community. We suggest that tES effects are moderated by pre-existing baseline E/I. Imbalances in E/I can be found in clinical or neuropsychiatric populations, during hormonal fluctuations (especially in women) and caused by interactions with the baseline neuronal activity, external influences, such as smoking or medication use, developmentally and age-related changes in E/I across the lifespan. Additional factors include individual differences in skull and cortical morphology, circadian influences that are currently not clarified, such as time of day or sleep deprivation, interactions with other neurotransmitter systems, and differential effects of tES due to unusual use of strategies in e.g., cognition. In addition, the current state of brain functioning and previous experiences can influence, or even flip polarity-dependent tDCS effects. For future research, it is of particular importance that scientists are aware of such variations and that they select their desired research populations with care in regard to potential unwanted noise in the data, and/or in some extreme cases the potential increase in seizure risk. These could be achieved by taking some of these factors into account, pre-assessing E/I levels or activation patterns using neuroimaging methods, in certain cases TMS, or at least behavioral patterns of performance.

## Conflict of interest statement

The authors declare that the research was conducted in the absence of any commercial or financial relationships that could be construed as a potential conflict of interest.
